# Causative organisms of post-traumatic endophthalmitis: a 20-year retrospective study

**DOI:** 10.1186/1471-2415-14-34

**Published:** 2014-03-25

**Authors:** Chongde Long, Bingqian Liu, Chaochao Xu, Yuan Jing, Zhaohui Yuan, Xiaofeng Lin

**Affiliations:** 1 State Key Laboratory of Ophthalmology, Zhongshan Ophthalmic Center, Sun Yat-sen University, 54 South Xianlie Road, Guangzhou, Guangdong 510060, China

**Keywords:** Endophthalmitis, Ocular trauma, Pathogens, Bacteria, Fungi, Susceptibility

## Abstract

**Background:**

A wide range of organisms that enter the eye following ocular trauma can cause endophthalmitis. This study was to investigate the spectrum of pathogens and antibiotic susceptibility of bacterial isolates from a large cohort of post-traumatic endophthalmitis cases.

**Methods:**

A retrospective study of 912 post-traumatic endophthalmitis patients treated at a tertiary eye-care center in China was performed. The associations between risk factors and the most common isolated organisms were investigated by Chi square Test. The percent susceptibilities for the first 10 years (1990–1999) and the second 10 years (2000–2009) were compared by Chi square test. p < 0.05 was considered statistically significant.

**Results:**

Three-hundred-forty-seven (38.1%) cases of endophthalmitis were culture-positive, and 11 (3.2%) showed mixed infections (Gram-negative bacilli and fungi), yielding a total of 358 microbial pathogens. Culture proven organisms included 150 (41.9%) Gram-positive cocci, 104 (29.1%) Gram-negative bacilli, 44 (12.3%) Gram-positive bacilli, and 60 (16.8%) fungi. The coagulase-negative staphylococcal (CNS) species *S. epidermidis* (21.8%) *and S. saprophyticus* (12.0%) were the predominant pathogens, followed by *Bacillus subtilis* (8.7%), *Pseudomonas aeruginosa* (7.8%), and *Escherichia coli* (6.4%)*.* Delayed repair over 24 h (p < 0.001) and metallic injury (p < 0.01) were significantly associated with positive culture of CNS. The most frequent fungal species were *Aspergillus* (26/60), followed by yeast-like fungi (18/60). *P. aeruginosa* was relatively sensitive to ciprofloxacin (83.3%), cefoperazone (75%), tobramycin (75%), cefuroxime (75%), and ceftazidime (75%) during the second decade. Multi-drug resistance was observed in the predominant Gram-negative bacteria.

**Conclusion:**

We identified a broad spectrum of microbes causing post-traumatic endophthalmitis, with Gram-positive cocci the most frequently identified causative organism, followed by *Bacillus* species, fungi, and mixed infections. CNS infection was statistically associated with delayed repair and metallic injury. Variation in antibiotic susceptibility was observed among isolated bacteria and between different periods. Ciprofloxacin and ceftazidime in the first and second decades of the study, respectively, showed the highest activity against bacterial post-traumatic endophthalmitis. For infections caused by *P. aeruginosa*, a combination therapy of ciprofloxacin, tobramycin, and one of the cephalosporins might provide optimal coverage according to data from the second decade.

## Background

Endophthalmitis following open-globe injury is one of the most devastating complications. The incidence rate of post-traumatic endophthalmitis was reported from 0 ~ 16.5% [1]. A wide range of microbes that enter the eye following ocular trauma can cause infective endophthalmitis. The microorganisms are derived from either the normal flora around the eyelid area, and gain entry after a delay in primary wound closure, or being carried into the wound by contaminated injury-causing objects [1-7]. The reported spectrum of causative organisms varies depending on the geographical location, type of injury, living environment, and time from injury to wound repair [1-3,8-14]. Most reports of post-traumatic endophthalmitis were described in case series of smaller numbers [1,9,13,15-17] and the isolated organisms showed variations in antibiotic susceptibility [8,9,12,18].

An understanding of the spectrum of microorganisms responsible for post-traumatic endophthalmitis is essential to guide empirical treatment. The purpose of this study was to investigate the spectrum of isolated microbes responsible for the development of post-traumatic endophthalmitis in cases presenting at one of the biggest tertiary eye care referral centers in southern China.

## Methods

This study was conducted in compliance with the principles of the Declaration of Helsinki and was approved by the ethics committee of Zhongshan Ophthalmic Center, Sun Yat-sen University. A retrospective analysis was performed on 912 cases presenting with post-traumatic endophthalmitis at Zhongshan Ophthalmic Center, Guangzhou, China, from January 1990 to December 2009. The diagnosis of post-traumatic endophthalmitis was based on the presence of worsening vision and pain, hypopyon, and vitritis following open-globe injuries or ocular penetrations. A sample of intraocular fluid was collected under sterile conditions from all patients with post-traumatic endophthalmitis presenting to our referral center. Anterior chamber fluids were aspirated through the limbus using a needle on a 1 ml plastic sterile disposable syringe. Vitreous specimens were obtained through the pars plana prior to antibiotic injection or vitrectomy. Smears were prepared for Gram- and Giemsa staining. Both anterior chamber taps and vitreous taps were collected in most of the cohort. When the vitritis was not serious in patients with hypopyon and intact lens capsule, only anterior chamber taps were taken. The aspirated intraocular fluids were immediately inoculated onto potato-sucrose agar for fungal culture, and onto blood agar and broth medium for bacterial culture, identification, and antibiotic susceptibility testing. Antibiotic susceptibility testing of isolated bacteria was performed using the traditional disc diffusion method.

The associations between risk factors and the most common isolated organisms were investigated using Chi square Test (Microsoft Office Excel 2003). The percent susceptibilities for the first 10 years (1990–1999) and the second 10 years (2000–2009) were also compared by Chi square analysis. p < 0.05 was considered statistically significant.

## Results

**Figure 1:**
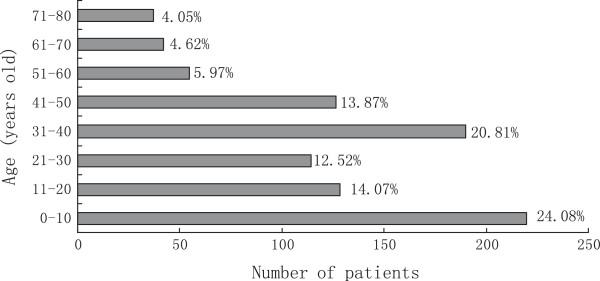
Demographics of the 912 cases of post-traumatic endophthalmitis.

During the 20-year study period, samples from 912 patients (774 male, 138 female) presenting with post-traumatic endophthalmitis were collected and subjected to microbiological analysis (mean age ± SD, 32.5 ± 20.1 years; range, 2–80 years). The demographic analysis of the cohort is shown in Figure 1. Patients younger than 11 years old composed 24.1% of the cohort, while patients aged from 31–40 years composed 20.8% of the total cohort. Of the 912 patients, 892 (97.8%) presented with open globe injuries and 20 presented with self-sealing wounds with a history of ocular penetration (exact location of injury unknown). Samples from 197 (21.6%) patients were smear positive, and 347 (38.0%) were culture positive for microbial growth. An interval between injury to primary repair of >24 h was found in 308/912 (33.8%) patients. Lens capsule rupture was present in 521/912 (57.1%) cases, while primary intraocular lens implantation was found in 38/912 (4.3%) patients. Intraocular foreign body (IOFB) was proved in 398 (43.6%) cases.

Table 1 summarizes the total number of microbes identified by culture and smear analysis. By sample culture, Gram-positive cocci were found to be the predominant cause of post-traumatic endophthalmitis (41.9%), followed by Gram-negative bacilli (29.1%), Gram-positive bacilli (12.3%), *Aspergillus* (7.3%), and yeast-like fungi (5.0%). Interestingly, we found 11 cases of mixed infection, all of which contained Gram-negative bacteria and fungi. There were 67 smear-positive and culture-negative cases, 122 smear-positive and culture-positive cases, and 225 culture-positive and smear-negative cases.

**Table 1:** Microorganisms identified by culture and smear

**Organisms**		**Culture (+) (n = 358)**	**Smear (+) (n = 197)**	**Total (n = 414)**
Bacteria	Gram(+) Cocci	150 (41.9%)	79 (40.1%)	190 (45.9%)
	Gram(-) Bacilli	104 (29.1%)	45 (22.8%)	120 (29.0%)
	Gram(+) Bacilli	44 (12.3%)	20 (10.2%)	55 (13.3%)
Fungi	Aspergillus	26 (7.3%)	25 (12.7%)	29 (7.0%)
	Yeast-like fungi	18 (5.0%)	16 (8.1%)	18 (4.3%)
	Other fungi	16 (4.5%)	12 (6.1%)	13 (3.1%)
Mixed infections	Gram(-) & fungi	11 (3.1%)	11 (5.6%)	11 (2.7%)

Table 2 shows the microbes isolated by specimen cultivation from post-traumatic endophthalmitis patients. Coagulase-negative *Staphylococcus* (CNS) species *S. epidermidis* (21.8%) and *S. saprophyticus* (12.0%) were the predominant bacterial species (33.8%), followed by *Bacillus subtilis* (8.7%), *Pseudomonas aeruginosa* (7.8%), *Escherichia coli* (6.4%), and *Staphylococcus haemolyticus* (5.3%)*.*

**Table 2:** Organisms isolated by culture from post-traumatic endophthalmitis patient samples

**Species**	**Organisms**	**No.**	**Percentage**
Gram(+) cocci	*Staphylococcus epidermidis*	78	21.8%
	*Staphylococcus saprophyticus*	43	12.0%
	*Staphylococcus haemolyticus*	19	5.3%
	*Staphylococcus aureus*	6	1.7%
	*Streptococcus liquefaciens*	1	0.3%
	Other Gram(+) cocci	3	0.8%
Gram(-) Bacilli	*Pseudomonas aeruginosa*	28	7.8%
	*Escherichia coli*	23	6.4%
	*Bacillus proteus*	16	4.5%
	*Acinetobacter lwoffii*	5	1.4%
	*Klebsiella pneumoniae*	4	1.1%
	*Enterobacter cloacae*	4	1.1%
	*Morgan bacillus*	3	0.8%
	*Bacillus alcaligenes*	3	0.8%
	*Stenotrophomonas maltophilia*	2	0.6%
	Other Gram(-) bacilli	16	4.5%
Gram(+) Bacilli	*Bacillus subtilis*	31	8.7%
	*Corynebacterium pyogenes*	2	0.6%
	Other Gram(+) bacilli	11	3.1%
*Aspergillus*	*Aspergillus fumigatus*	11	3.1%
	*Aspergillus nidulans*	7	2.0%
	*Aspergillus niger*	6	1.7%
	*Aspergillus flavus*	2	0.6%
*Yeast-like fungi*		18	5.0%
*Fusarium*	*Fusarium solani*	5	1.4%
	*Fusarium equiseti*	1	0.3%
	*Fusarium moniliforme*	1	0.3%
Other fungi	*Bipolaris sorodiniana*	4	1.1%
	*Curvularia geniculata*	2	0.6%
	*Conidia*	2	0.6%
	*Penicillium*	1	0.3%

**Figure 2:**
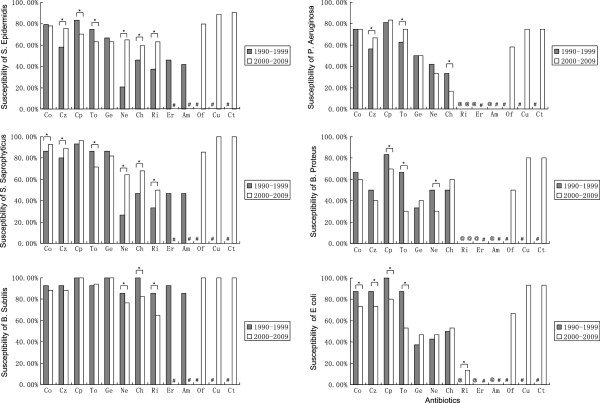
**Antibiotic susceptibilities of predominantly isolated bacteria.** Percent susceptibilities of predominantly isolated bacteria in the first ten-year period (1990–1999) and the second ten-year period (2000–2009) were compared by Chi square test. Co: cefoperazone, Cz: cefazolin, Cp: ciprofloxacin, To: tobramycin, Ge: gentamicin, Ne: neomycin, Ch: chloromycetin, Ri: rifampicin, Er: erythromycin, Am: ampicillin, Of: ofloxacin, Cu: cefuroxime, Ct: ceftazidime. #: antibiotics that were not tested. @: susceptibility was 0.00%. *: p < 0.01.

The relationships between positive culture of CNS and different variables (sex, age, retention of IOFB, composition of injury-causing object, lens capsule rupture, time between injury and repair) are listed in Table 3. Delayed repair over 24 h (p < 0.001) and metallic injury (p < 0.01) were both significantly associated with positive culture of CNS, while the other factors examined were not (p > 0.05). During the first decade, susceptibility of isolated bacteria to cefoperazone, cefazolin, ciprofloxacin, tobramycin, gentamicin, neomycin, chloromycetin, rifampicin, erythromycin, and ampicillin was tested, while during the second decade, ofloxacin, cefuroxime, and ceftazidime were included but erythromycin and ampicillin were omitted. Figure 2 illustrates the antibiotic susceptibilities of the predominant bacteria during the first and the second decades.

**Table 3:** **Relationship between positive culture of coagulase-negative staphylococci (*S. epidermidis and S. saprophyticus, CNS*) and variables**

**Variable**	**No. (%) of patients with positive CNS**	***P value***
Sex		
Male (n = 305)	102 (34.3%)	0.133
Female (n = 42)	19 (45.2%)	
Age		
10 or younger (n = 89)	35 (39.3%)	0.306
Older than 10 (n = 258)	86 (33.3%)	
Retained IOFB		
Yes (n = 172)	58 (33.7%)	0.656
No (n = 175)	63 (36.0%)	
Composition of injury-causing object		
Metallic injury (n = 213)	86 (40.4%)	0.007
Non-metallic injury (n = 134)	35 (26.1%)	
Time from injury to repair		
> 24 Hour (n = 94)	46 (48.9%)	0.001
≤ 24 Hour (n = 253)	75 (29.6%)	
Lens capsule rupture		
Yes (n = 201)	64 (31.8%)	0.165
No (n = 146)	57 (39.0%)	

*S. epidermidis* showed the greatest level of susceptibility to ceftazidime (90.7%), followed by cefuroxime (88.9%), but showed at least some resistance to all other antibiotics tested. *S. saprophyticus* was highly susceptible to ceftazidime (100%) and cefuroxime (100%), followed by ciprofloxacin (from 93.3% to 96.4%, p > 0.05). *B. subtilis* showed susceptibility (100%) to ciprofloxacin, gentamicin, ofloxacin, cefuroxime, and ceftazidime. *P. aeruginosa* showed high levels of resistance compared with other bacteria, especially to chloromycetin, the susceptibility to chloromycetin was 33.3% and 16.7% in the first and second decade, respectively (p < 0.001). *P. aeruginosa* was susceptible to ciprofloxacin, cefoperazone, cefuroxime, tobramycin, and ceftazidime (susceptibility range, 75%–83.3%) during the second decade. *B. proteus* showed 80% susceptibility to both cefuroxime and ceftazidime, while there was a significant decrease in susceptibility to tobramycin (from 66.7% to 30%, p < 0.001) and to neomycin (from 50% to 30%, p < 0.001) between the two decades. *E. coli* was susceptible to both cefuroxime and ceftazidime (93.3%), but its susceptibility to ciprofloxacin and tobramycin decreased by 20% (from 100% to 80%, p < 0.001) and 34.2% (from 87.5% to 53.3%, p < 0.001), respectively, between the two decades. Overall, ciprofloxacin showed the highest activity against all bacterial causes of post-endophthalmitis during the first decade, while ceftazidime showed the highest activity during the second decade.

## Discussion

Identification of causative pathogens is an important step in the management of infective endophthalmitis. A total of 358 culture-positive isolates were identified from 912 cases of post-traumatic endophthalmitis over 20 years. Of the 912 cases, 398 (44.6%) presented with an IOFB in our study. The presence of an IOFB (inert or non-inert) increases the incidence of post-traumatic endophthalmitis because it may be contaminated with infectious material [1,2,5,6,19]. *Staphylococcus* species were the most common infectious organisms, representing 40.8% (146/358) of overall isolates, followed by Gram-negative bacilli species (29.1%, 104/358, mixed infections included). *S. epidermidis* was the most common causative pathogen in this study, which agrees with findings from previous reports [9,10,12,13,17,20]. Al-Omran et al. [10]*showed that S. epidermidis* accounted for 37.1% of isolates in the group with IOFBs and 16.3% of isolates in the group without IOFBs (p < 0.05), indicating that IOFBs are positively associated with *S. epidermidis*. Out data showed that metallic injury and delayed repair are significantly associated with CNS (*S. epidermidis and S. saprophyticus*) infection. Interestingly, *S. saprophyticus* was a common pathogen, which has not previously been reported in post-traumatic ophthalmitis. We have observed that both *S. epidermidis* and *S. saprophyticus* were more frequently cultured from conjunctiva and eyelid margin smears compared with intraocular samples in patients with endophthalmitis (unpublished observation), indicating that there is a high possibility of these species penetrating through open wounds, especially in cases with delayed wound closure and injury caused by a metallic wire. There was also a relatively high incidence of Gram-negative bacilli associated with infection in our study, which has also been reported previously [8,10,11,18,21], though disagrees with findings from a study by Abu el-Asrar et al. [20]. In addition to predominantly-isolated CNS species and bacilli, we identified sporadic cases of *Acinetobacter lwoffii*, *Klebsiella pneumoniae*, *Enterobacter cloacae*, Morgan’s bacillus, *Bacillus alcaligenes*, *Corynebacterium pyogenes* (Gram-positive), *Stenotrophomonas maltophilia*, and *Staphylococcus liquefaciens* (Gram-positive), most of which (21/24) are virulent Gram-negative bacteria (Table 2). Because of the high incidence and urgent onset of bacillus endophthalmitis most authors recommended the use of prophylactic broad-spectrum intravitreal antibiotics to specifically cover *Bacillus* species especially in cases of open globe injury in a rural setting [22].

Post-traumatic endophthalmitis caused by a fungus is less common than bacterial cases, and are mainly found in open-globe injures with vegetable matter or soil contamination with a chronic onset [23]. In our study, 60 cases of fungal endophthalmitis were identified, of which 11 were mixed infections with concurrent Gram-negative bacterial infection. This pattern is similar to reports by Jindal et al. from India [24]. *Aspergillus* (8.4%, 26/347) was the most common fungal species [8,18,25,26], followed by yeast-like organisms (5.2%, 18/347) [8]. In addition to the predominant *Aspergillus* species (*A. fumigatus*, *A. nidulans*, *A. niger*, and *A. flavus*), sporadic cases of *Fusarium solani*, *Bipolaris sorodiniana*, *Curvularia geniculata*, *Fusarium equiseti*, *Penicillium*, *Fusarium moniliforme*, and *Conidia* were also identified (n = 15). Fungi constituted 60 (17.3%) of 347 isolates, similar to post-traumatic studies carried out in southern Florida [27] and in India [18]. This further supports the theory that fungal endophthalmitis cases resulting from trauma are primarily driven by tropical climate.

A positive culture results does not always agree with the clinical observation [1,12,28]. A positive result depends on the method of sample collection and inoculation, previous medical therapy, and culture medium. Many factors might have contributed to the relatively low recovery rate observed in the current study, the most likely factor being that the patients were treated with intravitreal, periocular, and systematic antibiotics prior to the collection of intraocular samples [1,13,29-31]. Smear tests might be more informative for microbe identification by Gram- and Giemsa staining. In the current study, staining methods positively identified 67 cases of culture-negative bacterial endophthalmitis (Table 1). Another benefit of smear testing is that its positive identification rate was very similar to that of culture methods for fungi (53 of 60), thus providing evidence for antifungal therapy.

Our antibiotic susceptibility analysis showed variation among the isolates and between the different time periods. During the first decade (1990–1999), ciprofloxacin was the most effective antibiotic against bacterial isolates, followed by cefoperazone. For the second decade (2000–2009), ceftazidime showed the greatest level of activity against most bacterial isolates, followed by cefuroxime. Neomycin showed little activity against most bacterial isolates, except *B. subtilis*, which was highly sensitive to all the tested antibiotics except erythromycin and ampicillin. Multi-drug resistance was observed in several Gram-negative organisms (*P. aeruginosa, B. proteus, and E. coli*) to all the tested antibiotics, especially gentamicin, neomycin, chloromycetin, and ofloxacin*.* These findings suggest that combination therapy could provide broader coverage against infection before susceptibility testing results were available. Systemic broad-spectrum antibiotic therapy is a common approach in the prophylaxis of bacterial endophthalmitis. Ariyasu et al. [32] demonstrated microbial contamination of the anterior chamber at the time of repair in one third of the open globe injury cases they studied. None of the patients developed endophthalmitis, indicating that the prophylactic use of antibiotics could reduce the incidence of endophthalmitis. However, Abu el-Asrar et al. [20] indicated that prophylactic antimicrobial therapy did not completely prevent bacterial infection. The indiscriminate, prolonged use of a wide range of antibiotics following open globe injury may be a major factor in the development of drug resistance among normal flora of the extra-ocular surface [12,33].

Despite the size of this cohort, which is the largest to date [8,14], this study has certain limitations, mainly as a result of its retrospective nature, including: 1) as a hospital-based study, selection bias might exist in our population of referred patients, who may represent more severe cases of infection. In addition, our database does not contain all patients with post-traumatic endophthalmitis as some patients were not followed-up after globe repair; 2) some of the isolated bacteria (16 Gram-negative bacilli and three Gram-positive cocci) could not be identified (Table 2) because of technical limitations. Culture of anaerobic bacteria was not performed, and we speculate that some of the smear test positive samples might include anaerobic bacteria; 3) susceptibility results were not available for vancomycin, although vancomycin covers most Gram-positive bacteria and has been widely used in recent years [17,24,34]; and 4) more information, such as degree of wound contamination, ocular trauma scoring, and intravitreal or periocular antimicrobial therapy prior to ocular sampling, was not available because some of the patients were transferred to our tertiary center following primary treatment in local hospitals.

## Conclusions

Overall, our study demonstrates a broad microbiological spectrum of post-traumatic endophthalmitis from a large-sized cohort in southern China over a 20-year period, with Gram-positive cocci being the most frequently identified organisms, followed by *Bacillus* species and fungi. CNS infection was statistically associated with delayed repair and metallic injury. Multi-drug resistance was observed in the predominant Gram-negative bacteria, and variation in susceptibility existed among isolated bacteria and between different periods. Ciprofloxacin in the first and ceftazidime in the second decade showed the highest activity against bacterial post-endophthalmitis. For endophthalmitis caused by *P. aeruginosa*, combination therapy with ciprofloxacin, tobramycin, and one of the cephalosporins might provide optimal coverage according to data from the period 2000–2009.

## Competing interests

The authors declare that they have no competing interests.

## Authors’ contributions

CL designed the study and participated in the data analysis. BL participated in the data analysis and drafted the manuscript. CX participated in the data collection. YJ participated in the data collection and analysis. ZY participated in the data analysis. XL participated in the design of the study. All authors read and approved the final manuscript.

## Pre-publication history

The pre-publication history for this paper can be accessed here:

http://www.biomedcentral.com/1471-2415/14/34/prepub
